# What Is the Optimal Strength Training Load to Improve Swimming Performance? A Randomized Trial of Male Competitive Swimmers

**DOI:** 10.3390/ijerph182211770

**Published:** 2021-11-10

**Authors:** Sofiene Amara, Emmet Crowley, Senda Sammoud, Yassine Negra, Raouf Hammami, Oussema Gaied Chortane, Riadh Khalifa, Sabri Gaied Chortane, Roland van den Tillaar

**Affiliations:** 1 Higher Institute of Sport and Physical Education of Ksar-Said, University of La Manouba, Tunis 2010, Tunisia; Coachsofieneamara@gmail.com (S.A.); senda.sammoud@gmail.com (S.S.); yassinenegra@hotmail.fr (Y.N.); raouf.cnmss@gmail.com (R.H.); oussama.gaeid@gmail.com (O.G.C.); riadhkhal@yahoo.fr (R.K.); sabrigaied1@gmail.com (S.G.C.); 2 Research Unit (UR17JS01) Sports Performance, Health & Society, Higher Institute of Sport and Physical Education of Ksar Saîd, University of La Manouba, Tunis 2010, Tunisia; 3 Biomechanics Research Unit, Department of Physical Education and Sport Sciences, University of Limerick, V94 T9PX Limerick, Ireland; crowleyperformance@gmail.com; 4 Laboratory of Cardio-Circulatory, Respiratory, Metabolic and Hormonal Adaptations to Muscular Exercise, Faculty of Medicine Ibn El Jazzar, Sousse 4002, Tunisia; 5 Department of Sport Sciences and Physical Education, Nord University, 7600 Levanger, Norway

**Keywords:** external training load, 1RM bench press, 1RM leg extension, start, turn

## Abstract

This study aimed to compare the effectiveness of high, moderate, and low resistance training volume-load of maximum strength training on muscle strength and swimming performance in competitive swimmers. Thirty-three male swimmers were randomly allocated to high (age = 16.5 ± 0.30 years), moderate (age = 16.1 ± 0.32 years) and a low resistance training volume-load group (age = 15.9 ± 0.31). This study was carried out in mid-season (January to March). Pre and post strength (e.g., repetition maximum [1RM] leg extension and bench press tests), swimming (25, 50 m front-crawl), start (speed, time, distance) and turn (time of turn) performance tests were conducted. Our findings revealed a large main effect of time for 1RM bench press: d = 1.38; 1RM leg extension: d = 1.55, and for 25 (d = 1.12), and 50 m (d = 1.97) front-crawl, similarly for start and turn performance (d = 1.28–1.46). However, no significant Group × Time interactions were shown in all strength swimming performances, start and turn tests (*p* > 0.05). In conclusion, low training loads have been shown to elicit the same results as moderate, and high training loads protocol. Therefore, this study shows evidence that the addition of low training volume-loads as a regular part of a maximal strength training regime will elicit improvements in strength and swimming performance.

## 1. Introduction

The Optimizing swimming performance involves improving the physical, physiological, and biomechanical parameters of swimming [1,2,3,4]. Several conditioning training methods and swimming training protocols have been shown to improve swimmers’ physical capacities (i.e., strength, power). Several studies argue that the effectiveness of the training protocols and methods depend on the event [5,6] and the intensity of the training sessions [7,8]. It has also been well established that swimming performance can be improved not only by in-water sport-specific training but also by means of dry land-training. More specifically, it has been reported that well developed levels of muscle strength and power play an important role in elite swimming performances [4,9,10,11]. For instance, strength training is an important component incorporated by coaches and swimmers into the swim training protocol for regional, national, and competitive levels [9,10,12]. Accordingly, various investigations [13,14] showed that, multiple objectives of the application of these forms of strength have been observed (i.e., metabolic adaptation, injury prevention and strength development).

Previous studies have examined several training modalities (i.e., strength training, and plyometric training) to improve the key factors of swimming performance [10,14]. Accordingly, many previous researchers have revealed the importance of upper and lower body strength on swimming performance. For example, Keiner et al. [15] indicated that the maximum strength of the upper and lower body is good predictor of performance in sprint swimming. West el al. [16] reported a strong correlation (r = 0.74) between the 1RM back squat and 15 m swimming performance. In addition, Morouço et al. [17] showed positive relationships between the tether swimming force parameters and front crawl swimming performance. The same authors [17] reported that the velocity of 50 m front crawl was strongly correlated with both maximum and average swimming forces (r = 0.76 and r = 0.81; *p* < 0.001, respectively) and maximum and average impulse (r = 0.91 and r = 0.70; *p* < 0.001, respectively). The bench press has been highlighted by elite strength and conditioning coaches as an important exercise that can improve upper body strength and ultimately swimming performance [4]. The primary muscles activated in bench press (the pectoralis major, the triceps brachii and the deltoid) are also activated by front crawl swimming [18,19]. For this reason, prescribing the bench press exercise can optimize the muscular propulsive forces during front crawl, and consequently improve swimming performance.

The lower body muscles play a crucial role in swimming performance, as several studies [11,12,20] have described the importance of lower body strength and power on swimming performance. These studies showed that strength and power were highly correlated with 25-m, and 50 m front crawl swimming performance [21,22]. Similarly, Thng et al. [23] indicated in their recently published review, that a range of outputs from different lower body dry-land resistance training exercises can be used to determine the lower body strength and power capacities of swimmers required for the swim start. These findings highlight the requirement for high levels of force and power to be developed through coordinated ankle, knee, and hip joint actions with those of the upper body to maximize take-off velocity [24]. Additionally, these findings highlight the need for multi-movement exercises to excel the transfer of strength training to swimming performance.

The prescription of maximum strength training also requires the consideration of training load, which can be categorized as either external or internal, where external training loads are objective measures of the work performed by the athlete (e.g., speed, acceleration, volume, training time, covered distance, or lifted weight) [24,25]. On the other hand, internal training load is defined as the relative physiological and psychological stressors imposed on the athlete during training or competition [26]. Various methods for measuring internal load exist, such as rating perceived exertion (RPE), session rating of perceived exertion (sRPE), training impulse (TRIMP), heart-rate indices, blood lactate, oxygen uptake and/or psychological scales and questionnaires [27]. The intensity of strength training is usually determined by the ratio of one repetition maximum (% of 1RM). However, the intensity of maximal strength training is generally set between 80% and 100% of 1RM [9,20]. In addition, total volume-load is determined by the number of repetitions and the number of sets per exercise [9] and different training protocols will have different total volume-load. For example, Strass [8], used a number of sets and repetitions ranged from one to three in a maximal strength training protocol for swimmers, and additionally, Girold et al. [9] prescribed three sets of six repetitions. On the contrary, Song et al. [28] prescribed similar sets but higher repetitions across a 24-week training intervention. (i.e., four phases: adaptation, maximum strength, power endurance and maximum strength and power/endurance). Therefore, Song et al. [28] would have a higher total-volume load compared to Strass. [8] and Girold et al. [9].

Obtaining the optimal volume training load needs careful planning and monitoring, to prevent both undertraining and overtraining [29], and an optimal balance between training and recovery must be maintained to maximize physical and specific performance. To the authors’ knowledge, the optimal maximal strength training strategy that may elicit the largest swimming performance improvements is still under debate. Given inconsistent findings in the literature on the effects of maximal strength training, as well as considerable heterogeneity across study characteristics and outcome measures (i.e., sample size, and age categories), further research is needed. Accordingly, we examine the effects of a 9-week maximal strength training of higher total volume-load, moderate total volume-load, and lower total volume-load on swimming performance. It was hypothesised that high training volume-loads would induce larger improvements on measures of swimming performance and muscular strength than moderate and low training volume-loads in swimmers.

## 2. Materials and Methods

### 2.1. Experimental Approach to the Problem

**Figure 1:**
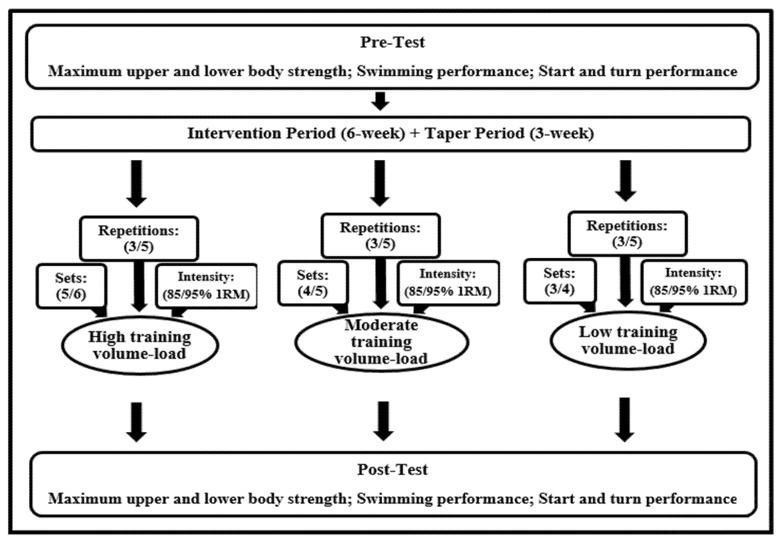
Description of the study design for the three groups.

A quasi-experimental research design was applied to address the research aim of the effect of high, moderate and low training volume-loads on maximal strength training in male swimmers. Participants completed an intervention period (6-weeks), followed by a taper period (3 weeks). Testing was conducted at before and after the whole period: swimming performance tests (25-m, 50 m front crawl), start (speed, time, distance), turn (time of turn) tests and strength tests (1RM in bench press and leg extension), were conducted. All tests were scheduled at least 48 h after the most recent training session or competition and under the same experimental conditions. The study design is presented in Figure 1.

### 2.2. Participants

Thirty-three male competitive swimmers participated in this study (Table 1). They were randomly allocated to HTVLG (*n* = 11, age = 16.5 ± 0.30 years, body-mass = 72.4 ± 5.3 kg, and height = 175 ± 9.8 cm), MTVLG (*n* = 11, age = 16.1 ± 0.32 years; body-mass = 73.5 ± 5.4 kg, height = 177 ± 9.7 cm) and LTVLG (*n* = 11, age = 15.9 ± 0.31, body-mass = 74.6 ± 5.5 kg, and height = 177 ± 9.4 cm). An a priori power analysis (G*Power 3.1.9.3) showed a total sample size of 21 (7 per group) was necessary to detect large effects (f = 0.80) using a power of 0.8 and alpha of 0.05. All participants competed on a national level, and they had a background of 7.5 ± 1.3 years of systematic swimming training involving seven to nine training sessions per week throughout the season. Swimmers had more than 4 years of resistance training where strength and conditioning training based on maximal strength was included for the last 2 years before the start of this study. Participants who missed more than 20% of the total training sessions and/or more than two consecutive sessions were excluded from the study. All participants and their parents/legal representatives were fully informed about the experimental protocol and its potential risks and benefits, and written consent was obtained from each participant before testing. The study was conducted in accordance with the Declaration of Helsinki, and the protocol was approved by the Ethics Committee of the Higher Institute of Sports and Physical Education of Ksar Saïd (UR17JS01).

### 2.3. Procedures

Details of the maximal strength training protocols are given in Table 2 for high, moderate, and low training volume-load. The protocols were conducted during the in-season period. The bench press and the leg extension were the only training exercises prescribed, to highlight transfer of exercises and remove other exercise bias. Before every maximal strength training session, a standardized 10 to 15 min warm-up was completed that included low intensity running, coordination exercises, dynamic movements (i.e., lunges and skips), sprints, and dynamic stretching for the higher and lower-limb muscles. Each training session lasts between 60 to 70 min. A total of three non-consecutive sessions per week were executed during the intervention period by each MST group. However, only two non-consecutive sessions per week were executed during the tapering period [30], see Table 2. All training protocols intensity varied between 85% 1RM and 95% 1RM, and the recovery between sets, and between exercises was set at 3 min in the three groups [8,9]. Two qualified instructors in strength and conditioning training supervised the protocols of dry land training.

In return, the swimmers followed their regular water-training program that consisted of seven sessions per week (between 4000 m and 6000 m per session). This training program in the water includes exercises of low to high intensity aerobic training, high intensity interval training and sprint training.

#### Monitoring

The quantification of training load was calculated in the two training periods (intervention and taper period) as follows: internal training load, rate of perceived exertion (RPE) score determined 30 min after each training session and external training load = total volume load was the sum of the volume load of the volume load of bench press and volume load of leg extension [31]. This was calculated by number of sets × number of repetitions × %1RM (sum of all volume load sessions per period) [25], see Table 3.

The 1RM strength test was conducted according to the protocol proposed by Negra et al. [32]. The leg press machine and Smith machine bench press were used to determine each subject’s maximal strength, to standardize technique across participants. Before attempting a 1RM trial, participants performed 5 to 6 repetitions with a relatively light load (40% of their estimated 1RM). Thereafter, 3 to 4 repetitions were performed with a heavier load (70% of their estimated 1RM). Finally, a single repetition was conducted with a load corresponding to 95% of the estimated 1RM. Participants then attempted a single repetition with the perceived 1RM load. If this load was lifted with the proper technique, the load was increased by another 1.0–2.5 kg, and the participant attempted another repetition. Failure was defined as a lift falling short of the full range of motion on at least two trials with a 2 min rest interval between trials. The 1RM was typically determined within 4 to 5 trials.

All swimming performance tests were conducted in a 25 m indoor pool with 27.1 and 25.9 °C of water and air temperatures, respectively, and 64% relative humidity. Before the start of the test all swimmers completed an 800 m warm-up (600 m aerobic swimming + 200 m progressive sprint swimming), applying the swimmer’s usual strategies. Two expert timekeepers recorded performance times using stopwatch (SEIKO S120-4030, Tokyo, Japan) noted in seconds. Swimmers performed two front crawl swimming tests (1st: 25-m, 2nd: 50 m font crawl that the recovery between tests was set at 5-min). The ICCs for Pre to Post-test reliability of 25 m and 50 m front crawl was included between 0.98 and 0.99.

The start and turn performances were determined during the 50 m front crawl [33]. The protocol consisted of filming all the 50 m test video sequences by two fixed side video cameras (Sony, SNC VB 603, Tokyo, Japan; 50 Hz, full HD, 1080 p) placed in the stands (7 m above and 7 m from the edge of the pool). Video analysis system software (Kinovea, version 0.8.15, Joan Charmant and Contrib., kinovea.org) [34] was used to determine the time of start (the time lag between the start signal and the heads contacting with water), the distance of start (the distance lag between the start signal and the heads contacting with water) and the time of turn (the time lag between the touch on the wall and the 5 m mark). The speed of start (the speed lag between the start signal and the heads contacting with water) was determined by the equation: Speed of start = Distance of start/Time of start [35]. The ICCs for pre to post-test reliability of all start and turn performances were included between 0.95 to 0.96.

### 2.4. Statistical Analyses

Data are presented as group mean values and standard deviations. After data normality was verified with the Shapiro–Wilk test, a one-way analysis of variance (ANOVA) was used to detect baseline between-group differences. To establish the effect of the interventions on the dependent variables, a three (group: high, moderate, and low training volume-load) × 2 (time: pre-test, and post-test) ANOVA with repeated measures was computed. Bonferroni post hoc procedure was applied to locate pairwise differences, only if a significant F-value was observed. Additionally, effect sizes (ES) were determined by converting partial eta-squared from the ANOVA output to Cohen’s d (0.00 < d < 0.49), medium (0.50 < d < 0.79), and large (d > 0.80) [36]. Test–retest reliability was assessed using ICCs [37]. Statistical analyses were carried out using the SPSS 22 (SPSS Inc., Chicago, IL, USA). Significance levels were set at α = 5%.

## 3. Results

All participants received treatment conditions as allocated. Adherence rate to training was 98%, for all groups. None of the participants reported any training- or test-related injuries. There were no statistical significances between-group baseline differences for age, height, and body-mass. Additionally, no significant between-group differences were recorded at pre-test regarding proxies of swimming performances, start, and turn and strength test.

The 1RM bench press results indicated a main effect of time (d = 1.38 [large], *p* < 0.001) with no training group × time interaction (d = 0.31 [small], *p* > 0.05). Regarding 1RM leg extension, similar results were observed with a main effect of time (d = 1.55 [large], *p* < 0.001) and no training group × Time interaction (d = 0.53 [medium], *p* > 0.05) (Table 4).

Significant main effects of time were found for the 25 m (d = 1.97 [large], *p* < 0.001) and 50 m (d = 1.12 [large], *p* < 0.001) front crawl. In addition, no significant group × time interactions were observed for the 25-m, and 50 m sprint performance (d = 0.18 [small], d = 0.28 [small], respectively. all *p* > 0.05) (Table 4).

Our statistical calculation revealed significant main effects of time of speed of start, time of start and distance of start (d = 1.30 [large], 1.29 [large], 1.28 [large], respectively, all *p* < 0.001). However, training group × time interactions failed to reach the significance level for all tests (d = 0.35 [small], 0.36 [small], 0.33 [small], respectively, all *p* > 0.05]. For the time of turn test, results revealed a significant main effect of time (d = 1.46 [large], *p* < 0.001). No training group × time interaction was observed (d = 0.40 [small], *p* > 0.05) (Table 4).

## 4. Discussion

To the authors’ knowledge, this is the first study to examine and compare the effects of three different training volume-loads of maximal strength training protocols on muscle strength and swimming performance in national level swimmers. The findings of this study showed that the different training loads of maxima strength training protocols were effective in improving swimming performance in male swimmers.

Strength performance is a key performance determinant in swimming. Our results revealed significant improvements in the 1RM leg extension and bench press tests after 9 weeks of maximal strength training. This agrees with previous investigations that observed increases in strength performance after maximal strength training intervention [9,10,12]. For instance, Girold et al. [9] showed significant improvements in peak torque after four weeks of maximal strength training. Improvements were seen in the concentric condition at 60° s^−1^ (11.2 ± 13.6) and at 180° s^−1^ (16.9 ± 11.7%). The same authors [9] revealed significant improvements in the mean swimming velocity for 50 m front crawl (2 ± 1.3%) and in the stroke length (2.93%) at the end of the training period in national level of competitive swimmers (age = 21.8 years). Similarly, Girold et al. [10] reported significant improvements in muscle strength after 12 weeks of maximal strength training in the elbow flexors in the isometric condition (39.5 ± 32.4%) and in the elbow extensors in the isometric and concentric conditions at 60° s^−1^ and at 180° s^−1^ (45.5 ± 38.7%; 33.7 ± 27.6%; 35.2 ± 31.9%, respectively) and significant improvement in the performance of 50 m swimming (2.8 ± 2.5%). Additionally, Aspenes et al. [12] reported a significant increase in tethered maximal swimming force (6.9%), and the maximal strength in bilateral shoulder extension (20.3%) and significant improvements in the 400 m maximal front crawl swimming (1.40%) after 11 weeks of combined intervention of maximal strength training and high-intensity interval training.

Maximum muscle strength was found to be a good predictor of swimming performance [9,12,22,38]. Our findings found that all experimental groups demonstrated significant improvements in maximum muscle strength and swimming performance tests after lower, moderate, and higher doses of maximal strength training. These results do not confirm our hypothesis that only the high training volume-loads dose would induce larger improvements on measures of swimming performance and muscular strength than moderate and low training volume-loads in swimmers. The age and the resistance training experience exercised by the swimmers prior to this study may explain these results. As, Slimani et al. [39] had shown in a meta-analysis that strength training could improve muscle power in the lower limbs regardless of the training doses applied. The same authors [39] found that longer training times (>8 weeks) are more effective in improving lower limb power in young athletes (age: 12 to 18 years). Our results are in line with those established by Kubo et al. [38], who demonstrated that 10-weeks of three different training load dose of maximal strength training protocols (higher load–lower repetition: 4RM for seven sets; intermediate load–intermediate repetition: 8RM for four sets; lower load–higher repetition: 12RM for three sets) improved maximum muscle strength in bench press test (26.6%, 27.8%, 17.9%, respectively) in healthy men (19.5 ± 24.0 years). Furthermore, Paterson et al. [40], recommended that the dose of maximal strength training should be characterized by a mean training intensity of 85% of 1RM, two sessions per week, and with a mean training volume of eight sets per muscle group across the week.

Although a study elaborated by Keiner et al. [14] has shown that there is a correlation between the 1RM bench and starting performance, our study presents the first investigation that studies the effect of maximum strength training on starting and turning performance. Our results showed that MST training with different loads could improve start and turn performance. Our results are in line with the study by Bishop et al. [34]. However, the same authors [34] had shown that a dry land based in plyometric training for eight weeks could improve the starting speed (14.73%) in competitive adolescent swimmers (13.1 ± 1.4 years). In addition, Potdevin et al. [41] revealed that 6 weeks of combined swimming and plyometric training could improve start and turn performance in pubescent swimmers (age = 14.3 ± 0.2 years).

This study has some limitations that warrant discussion. Firstly, the movement velocity of strength and conditioning exercise was not controlled by the supervised instructors during all training protocols. Secondly, we were only able to assess performance but not physiological data, which is why we cannot provide evidence on the underlying neuromuscular mechanisms responsible for the observed findings. Another limitation is the experience of strength training before the intervention (learning effect of the test). It has been shown that a low training volume in relatively young athletes is enough to increase strength and swimming performance. For more experienced athletes this could be different and must be studied in those groups. Therefore, the present findings are only related to swimmers of this age/experience. Future studies could examine the effect of maximum strength training with different loads on swimming kinematic variables. In addition, other future studies could shed light on the concept of the transformation from maximum strength gain to propulsive swimming in water in competitive swimmers.

## 5. Conclusions

Findings of this study suggest that the different maximal strength training protocols are safe (i.e., no injuries occurred), and feasible (2 to 3 training sessions per week) in adolescent male swimmers. The different load of strength training protocols all seem to be beneficial to improve swimming performance and, therefore, it can be suggested that minimal dose of MST is required to elicit performance enhancements in 1RM muscle strength of upper and lower body and swimming performance tests. Accordingly, the minimal dose of low training volume-load have been shown to elicit the same results as moderate and high training volume-load protocols. Based on the findings reported herein, strength and conditioning trainers should consider including low training volume-load as a regular part of the strength training regime in adolescent male swimmers to promote physical fitness and ultimately improve swimming performance.

## Figures and Tables

**Table 1:** Main characteristics of the competitive swimmers.

Characteristics	HTVL_G_ (*n* = 11)	MTVL_G_ (*n* = 11)	LTVL_G_ (*n* = 11)
Age (y)	16.5 ± 0.30	16.1 ± 0.32	15.9 ± 0.31
Height (cm)	175 ± 9.80	177 ± 9.70	177 ± 9.40
Body mass (kg)	72.4 ± 5.3	3.3 ± 0.25	74.6 ± 5.5
Swimming experience (y)	8.70 ± 0.29	8.63 ± 0.27	8.43 ± 0.25
Resistance experience (y)	4.2 ± 0.23	4.3 ± 0.25	4.5 ± 0.24

**Table 2:** Detailed description of the 9-week MST in the three groups.

			Training Volume Load Group		
Period	Week (Session)	Exercises	High	Moderate	Low
Intervention period	W1 (S1/S2/S3)	BP	5 × (5 × 85% 1RM)	4 × (4 × 85% 1RM)	4 × (3 × 85% 1RM)
		LE	5 × (5 × 85% 1RM)	4 × (4 × 85% 1RM)	4 × (3 × 85% 1RM)
	W2 (S4/S5/S6)	BP	5 × (4 × 90% 1RM)	4 × (4 × 90% 1RM)	4 × (3 × 90% 1RM)
		LE	5 × (4 × 90% 1RM)	4 × (4 × 90% 1RM)	4 × (3 × 90% 1RM)
	W3 (S7/S8/S9)	BP	5 × (3 × 95% 1RM)	4 × (3 × 95% 1RM)	4 × (3 × 95% 1RM)
		LE	5 × (3 × 95% 1RM)	4 × (3 × 95% 1RM)	4 × (3 × 95% 1RM)
	W4 (S10/S11/S12)	BP	6 × (3 × 95% 1RM)	5 × (3 × 95% 1RM)	4 × (3 × 95% 1RM)
		LE	6 × (3 × 95% 1RM)	5 × (3 × 95% 1RM)	4 × (3 × 95% 1RM)
	W5 (S13/S14/S15)	BP	6 × (4 × 90% 1RM)	5 × (4 × 90% 1RM)	4 × (4 × 90% 1RM)
		LE	6 × (4 × 90% 1RM)	5 × (4 × 90% 1RM)	4 × (4 × 90% 1RM)
	W6 (S16/S17/S18)	BP	6 × (5 × 85% 1RM)	5 × (5 × 85% 1RM)	4 × (4 × 85% 1RM)
		LE	6 × (5 × 85% 1RM)	5 × (5 × 85% 1RM)	4 × (4 × 85% 1RM)
Taper period	W7 (S19/S20)	BP	5 × (5 × 85% 1RM)	4 × (5 × 85% 1RM)	4 × (4 × 90% 1RM)
		LE	5 × (5 × 85% 1RM)	4 × (5 × 85% 1RM)	4 × (4 × 90% 1RM)
	W8 (S21/S22)	BP	5 × (4 × 90% 1RM)	4 × (4 × 90% 1RM)	3 × (5 × 85% 1RM)
		LE	5 × (4 × 90% 1RM)	4 × (4 × 90% 1RM)	3 × (5 × 85% 1RM)
	W9 (S23/S24)	BP	5 × (3 × 95% 1RM)	4 × (3 × 95% 1RM)	3 × (3 × 95% 1RM)
		LE	5 × (3 × 95% 1RM)	4 × (3 × 95% 1RM)	3 × (3 × 95% 1RM)

BP: bench press, LE: leg extension, RM: Repetition maximum, Example: 5 × (5 × 85% 1RM): 5 sets × 5 repetitions × 85% 1RM.

**Table 3:** Quantification of external and internal training load during the 9-week of MST.

			Training Volume-Load Group			
Period	Training Load		High	Moderate	Low	*p*-Value (ES)
Intervention period	External	Volume Load BP (kg)	16339 ±1386.48	13124 ± 985	10474 ± 865.52	<0.001 (4.57)
		Volume Load LE (kg)	17816 ± 1725.24	14366 ± 1191.35	10868 ± 998.14	<0.001 (4.44)
		Total Volume Load (kg)	34154 ± 3054.23	27490 ± 2159.78	21342 ± 1837.93	<0.001 (4.57)
	Internal	RPE	8.64 ± 0.54	6.82 ± 0.75	4.95 ± 0.69	<0.001 (4.63)
Taper period	External	Volume Load BP (kg)	4951.2 ± 420.15	4031 ± 302.53	3297.4 ± 272.4	<0.001 (4.20)
		Volume Load LE (kg)	5398.6 ± 522.80	4412.3 ± 365.91	3602.5 ± 330.8	<0.001 (3.71)
		Total Volume Load (kg)	10350 ± 925.52	8443.3 ± 663.36	6899.8 ± 595.0	<0.001 (3.99)
	internal	RPE	4.34 ± 0.55	3.46 ± 0.42	2.45 ± 0.48	<0.001 (3.20)

BP: bench press, LE: leg extension, ES: effect size.

**Table 4:** Group-specific pre-test and post-test performances after 9 weeks of an in-season maximal strength training on muscle strength and swimming-specific performance in competitive swimmers.

	Training Volume-Load Group							
Performances	High		Moderate		Low		*p*-Value (ES)	
	Pre-Test	Post-Test	Pre-Test	Post-Test	Pre-Test	Post-Test	Time	Group × Time
25 m front crawl (s)	13.52 ± 0.56	12.76 ± 0.54	13.55 ± 0.53	12.91 ± 0.54	13.56 ± 0.51	13.02 ± 0.52	<0.001 (1.27)	0.785 (0.18)
50 m front crawl (s)	26.91 ± 1.29	25.20 ± 1.26	26.92 ± 1.24	25.52 ± 1.24	26.94 ± 1.23	26.03 ± 1.23	<0.001 (1.13)	0.570 (0.28)
Speed of start (s)	3.06 ± 0.23	3.43 ± 0.21	3.06 ± 0.23	3.31 ± 0.21	3.09 ± 0.23	3.29 ± 0.22	<0.001 (1.30)	0.420 (0.35)
Time of start (s)	0.90 ± 0.04	0.84 ± 0.03	0.89 ± 0.04	0.85 ± 0.03	0.89 ± 0.04	0.85 ± 0.04	<0.001 (1.29)	0.466 (0.33)
Distance of start (m)	2.73 ± 0.09	2.87 ± 0.09	2.73 ± 0.08	2.81 ± 0.07	2.73 ± 0.08	2.80 ± 0.08	<0.001 (1.28)	0.378 (0.36)
Time of turn(s)	1.99 ± 0.04	1.92 ± 0.04	2.01 ± 0.04	1.96 ± 0.04	2.01 ± 0.04	1.96 ± 0.04	<0.001 (1.46)	0.299 (0.40)
1RM bench press (kg)	46.27 ± 3.93	52.64 ± 3.91	47.09 ± 3.53	51.55 ± 3.78	46.18 ± 3.82	50.00 ± 3.27	<0.001 (1.38)	0.501 (0.31)
1RM leg ext. (kg)	50.46 ± 4.90	60.46 ± 4.57	51.55 ± 4.28	56.64 ± 4.48	50.45 ± 4.63	55.55 ± 4.50	<0.001 (1.55)	0.128 (0.53)

RM: Repetition maximum; leg ext: leg extension; ES: Cohen’s d (effect size).

## Data Availability

The data presented in this study are available on reasonable request from the corresponding author.
